# Co-exposure to environmental carcinogens *in vivo* induces neoplasia-related hallmarks in low-genotoxicity events, even after removal of insult

**DOI:** 10.1038/s41598-018-21975-w

**Published:** 2018-02-26

**Authors:** Marta Martins, Ana Silva, Maria H. Costa, Célia Miguel, Pedro M. Costa

**Affiliations:** 1 MARE - Marine and Environmental Sciences Centre, Departamento de Ciências e Engenharia do Ambiente, Faculdade de Ciências e Tecnologia da Universidade Nova de Lisboa, 2829-516 Caparica, Portugal; 2 UCIBIO-REQUIMTE – Research Unit on Applied Molecular Biosciences, Departamento de Química, Faculdade de Ciências e Tecnologia da Universidade Nova de Lisboa, 2829-516 Caparica, Portugal; 3 Instituto de Biologia Experimental e Tecnológica (iBET), Apartado 12, 2781-901 Oeiras, Portugal; 4 Instituto de Tecnologia Química e Biológica António Xavier da Universidade Nova de Lisboa (ITQB NOVA), Av. República, 2780-157 Oeiras, Portugal; 5 Biosystems & Integrative Sciences Institute, Faculdade de Ciências da Universidade de Lisboa (FCUL), Campo Grande, 1749-016 Lisbon, Portugal; 6 UCIBIO-REQUIMTE – Research Unit on Applied Molecular Biosciences, Departamento de Ciências da Vida, Faculdade de Ciências e Tecnologia da Universidade Nova de Lisboa, 2829-516 Caparica, Portugal

## Abstract

Addressing the risk of mixed carcinogens *in vivo* under environmentally-realistic scenarios is still a challenge. Searching for adequate biomarkers of exposure requires understanding molecular pathways and their connection with neoplasia-related benchmark pathologies. Subjecting the zebrafish model to realistic concentrations of two genotoxicants and carcinogens, cadmium and benzo[a]pyrene, isolated and combined, yielded low levels of DNA damage. Altogether, the organisms’ mechanisms of DNA repair, oxidative stress and phases I and II were not overwhelmed after two weeks of treatment. Still, transcriptional responses related to detoxification (epoxide hydrolase and UDP-glucuronosyltransferase) were higher in animals subjected to the combination treatment, inclusively following depuration. Nonetheless, inflammation and formation of hyperplasic foci in fish epithelia were more severe in animals exposed to the combined substances, showing slower recovery during depuration. Additionally, the combination treatment yielded unexpected increased expression of a *ras*-family oncogene homologue after depuration, with evidence for increased tp53 counter-response in the same period. The findings indicate that oncogene expression, cell proliferation and inflammation, may not require noticeable DNA damage to occur. Furthermore, albeit absent proof for neoplasic growth, the removal of chemical insult may promote tissue recovery but does not entirely clear molecular and histopathological endpoints that are commonly associated to neoplasia.

## Introduction

Carcinogenesis induced by environmental factors, genotoxicants and mutagens included, is suspected to account for a very significant proportion of all neoplasic diseases in humans (and wildlife), probably attaining up to 19%^1^. However, environment quality guidelines are essentially based on single-toxicant assays and is hindered in its quest for plausible cause-effect relationships by the fact that toxicants seldom occur isolated in the environment. Additionally, customary constraints should be considered when dealing with more realistic circumstances of assessment, which include *in vivo* models, dose and route of exposure. In particular, in spite of much research in the mechanisms underlying the *modus operandi* of benchmark mutagens, such as polycyclic aromatic hydrocarbons and other Ahr (aryl hydrocarbon receptor) agonists, little is known on the molecular pathways that relate to oncogene activation and their relation to genotoxicity and damage control mechanisms under realistic circumstances. These damage control pathways include the ability to repair DNA damage, promote death of cells damaged beyond repair (therefore restraining diffusion of mutations) and control cell proliferation and inflammation, all of which are related to known hallmarks of cancer^2^.

The mutagenic and potentially carcinogenic pathways differ quite substantially between chemicals. Ahr agonists like benzo[a]pyrene (B[a]P) are hydrophobic and relatively inert until they are bioactivated by cytochrome P450 (CYP) mixed-function oxidases during phase I of detoxification, followed by action of epoxide hydrolases that will yield the highly mutagenic B[a]P-diol epoxides (BPDE). Non-essential metals like Cd, which is not even a Fenton metal and cannot therefore generate reactive oxygen species (ROS) on its own, is an indirect genotoxicant that disrupts the normal functioning of many enzymes, including those involved in DNA damage repair and detection^3^. It has also been demonstrated *in vitro* that Cd can modulate B[a]P metabolism^4^. Altogether, co-exposure to these two toxicants, which have become paradigmatic models for environmental toxicologists, mostly due to their potency and for being ubiquitous in the environment, may yield unexpected events that compromise drawing clear cause-effect relationships. Nonetheless, most research has been performed *in vitro*, with evident difficulties in transposing knowledge to *in vivo* models and, moreover, human health. The *in vivo* aspect is of particular relevance, as neoplasia is a whole-system process that implicates all sub-individual levels of biological organisation and requires time to occur that is usually not compatible with the duration of standardised short- to mid-term bioassays.

The constraints posed by toxicant mixtures and realistic exposure scenarios to toxicologists that study environmental carcinogens invariably have been leading to divergent views toward the biomarker and risk assessment concepts. In fact, genotoxicity can be little informative under these circumstances^5^, which may compromise its value as early-stage warning of exposure and risk. Original research on patterns of molecular responses as biomarkers through high-throughput “omics” methods is increasingly common^6^. Nonetheless, there is scarce information if and how measurable amounts of DNA damage can linearly relate to the risk of developing neoplasia and if carcinogens, isolated or combined, can actually increase this risk without necessarily exceeding the cells’ defences against insult. Indeed, current perspectives on the relationship between DNA damage and neoplasia do not highlight bulk genomic alteration as aetiological agent. Instead, the prevalence of mutation, or hypermutation, in localised sites, results in signatures of mutations that can be used as biomarkers, as may be linked to certain types and stages of cancer^7^. Another line of research upholds the discovery of gene expression signatures for the same purpose^8^. In spite of the recent advances in high-content screening methods upon which both approaches are built upon, the relationship between genotoxicity, mutation burden and transcriptome remains difficult, especially since the vast majority of mutations in human cancer cell lines are transient whereas only a few are known to be involved in aggressive proliferation of neoplasic cells^7–9^.

The present work aims at disclosing the potential effects of two distinct model carcinogens, Cd and B[a]P on the setting of neoplasia-related pathological features *in vivo* at pathological and molecular levels by integrating phase I and II responses with genotoxicity, oncogene activation and their immediate responses. The two substances are acknowledged as potential carcinogenic for human and fish, respectively, For the purpose, the zebrafish was selected as model, considering its high genomic annotation and its growing value as surrogate for mutagenesis and neoplasia-related research^10^. The primary goal was to ascertain if genotoxicity observed in zebrafish can be correlated with changes in molecular pathways of damage and response during exposure and after removal of insult and if these can be linked to actual histopathological markers.

## Results

### Mortality and gross pathology

Mortality was low and seemingly stochastic, being restricted to T_14_ (exposure time) for Cd and mixtures treatments (one casualty in each). All fish were sampled alive and no gross external pathology lesions or noticeable changes in the behavioural patterns were observed.

### Genotoxicity

**Figure 1:**
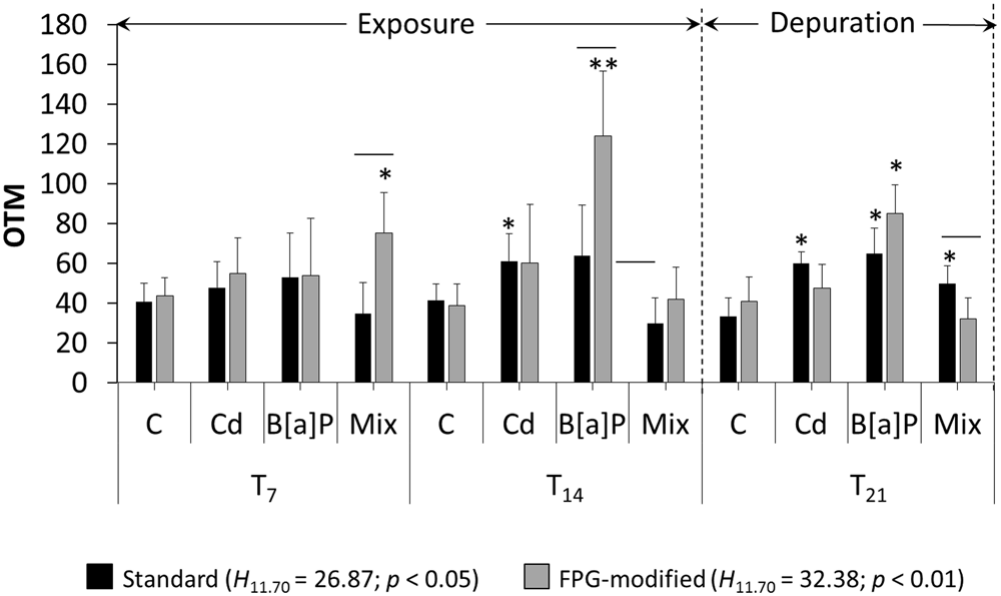
Determination of DNA damage through the Comet assay. Olive tail moment (OTM) mean values ± 95% confidence interval retrieved from the standard and FPG-modified protocols of the Comet assay for each experimental treatment (C – control; Cd – cadmium treatment (100 μg L^−1^), B[a]P – benzo[a]pyrene treatment (500 ng L^−1^); Mix – combined Cd and B[a]P (100 μg L^−1^ plus 500 ng L^−1^, respectively). Sampling times were at day 7 (T_7_) and 14 (T_14_) of the exposure period and at day 21 (T_21_), the end of the seven-day depuration. * and **represent significant differences to respective sampling time control, *p* < 0.05 and *p* < 0.01, respectively (Mann-Whitney *U* test). Horizontal bars indicate significant differences between standards and FPG-post treatment Comet assay (Wilcoxon Matched Pairs test, *p* < 0.05).

Significant variations in Olive Tail Moment (OTM) values were found between all treatments and sampling times, concerning both standard and FPG-modified Comet assay (Kruskal-Wallis *H* test, *p* < 0.05 and *p* < 0.01, respectively). During the contaminant exposure period, the B[a]P and mixture treatments yielded the highest induction of DNA strand breakage, at T_7_ and T_14_, respectively, after the FPG post-treatment, comparatively to experimental controls (Mann-Whitney *U* test, *p* < 0.05 and *p* < 0.01, respectively) and to standard Comet (Wilcoxon Matched Pairs test, *p* < 0.05), as presented in Fig. 1. Following the seven-day depuration (T_21_), all treatments revealed significantly higher OTM from the standard Comet assay, comparatively to controls (Mann-Whitney *U* test, *p* < 0.05). During this period, the FPG post-treatment cells presented the highest OTM value for B[a]P treatment.

Bilobed and budding nuclei were the most frequent nuclear abnormalities in red blood cells (RBC) present in fish subjected to any treatment (Supplementary Information (SI) Fig. S1). ENA scoring resulted in absence of significant differences between treatments (Kruskal-Wallis *H* test, *p* > 0.05), as displayed in Supplementary Fig. S2). Only fish subjected to the B[a]P test at T_7_ yielded significantly lower scores than controls (Mann-Whitney *U* test, *p* < 0.05). The percentage of reticulocytes was higher in comparison with leukocytes in all treatments (Supplementary Fig. S3), with the exception of fish exposed to Cd for 7 days (T_7_).

### Oxidative stress biomarkers

Significant differences in lipid peroxides (LPO) levels, catalase (CAT) activity and total antioxidant capacity (TAC) were recorded among treatments and sampling times (Kruskal-Wallis *H* test, *p* < 0.01, *p* < 0.05 and *p* = 0.01, respectively). During exposure, significant differences of LPO levels were recorded at T_7_ for B[a]P test (Mann-Whitney *U* test, *p* < 0.01), as figured in Supplementary Fig. S4A. Furthermore, fish subjected to the mixture showed significantly higher LPO levels comparatively to controls following depuration (Mann-Whitney *U* test, *p* < 0.05), albeit not during exposure. Catalase activity and TAC depicted a similar trend (Supplementary Fig. S4B and C, respectively): overall, the levels were lower than controls, regardless the treatment and the time of exposure.

### RT-PCR

**Figure 2:**
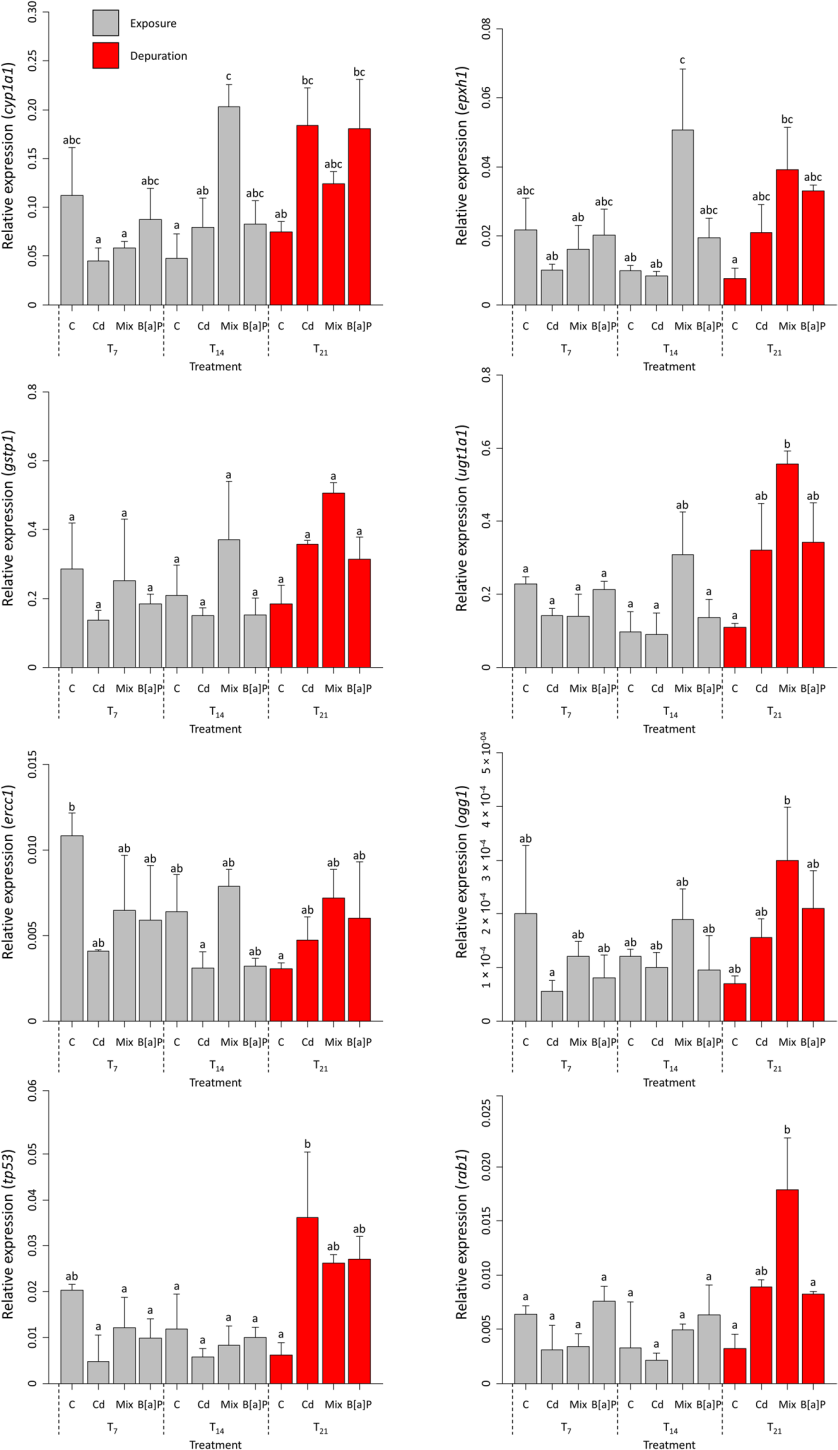
Expression of target genes. Gene expression plots for all experimental treatments (Cd, Mix and B[a]P), including the 14- and subsequent 7-day exposure and recovery (depuration), retrieved from hepatopancreas mRNAs (*n* = 2). Notice the somewhat similar trend during depuration, in particular, for most responses, and elevated *tp53* transcription at this time point relatively to the exposure period. Data are given as means ± standard deviation. Different letters indicate significant differences (Tukey HSD test, *p* < 0.05, performed on log-transformed data).

The expression of each target genes (see Fig. 2) was significantly modulated by the different experimental treatments and sampling times (*F* test, adjusted *p* < 0.05). In spite of the trend for increased gene expression in control T_7_ animals (i.e. exposed to vehicle only for a week), more obvious for *ercc1*, this effect was not statistically significant and was not recorded at latter stages of the experiment. Overall, the expression levels of *cyp1a1*, *gstp1* and *ugt1a1* were higher than those of the other genes by one or two orders of magnitude, regardless of treatment. The expression of these genes, together with *ephx1*, *ercc1* and *ogg1*, the latter two involved in DNA repair and the former in phase I metabolism, like *cyp1a1*, yielded a similar pattern, namely, the mixture of compounds caused the most significant increase in expression, especially of phase I-related genes, at T_14_. On the other hand, at both T_7_ and T_14_ (exposure time), Cd and B[a]P, isolated, did not change the expression of genes relatively to control. Conversely, following depuration (T_21_), the expression of most analysed genes increased for all treatments, relatively to controls, especially in the mixture treatment. The expression of *tp53* and the *rab1* oncogene was elevated only after the one-week depuration and not during exposure, in the latter case, attaining ≈7 fold, comparatively to controls, in animals exposed to the mixture.

### Gill histopathology and Immunohistochemistry

**Figure 3:**
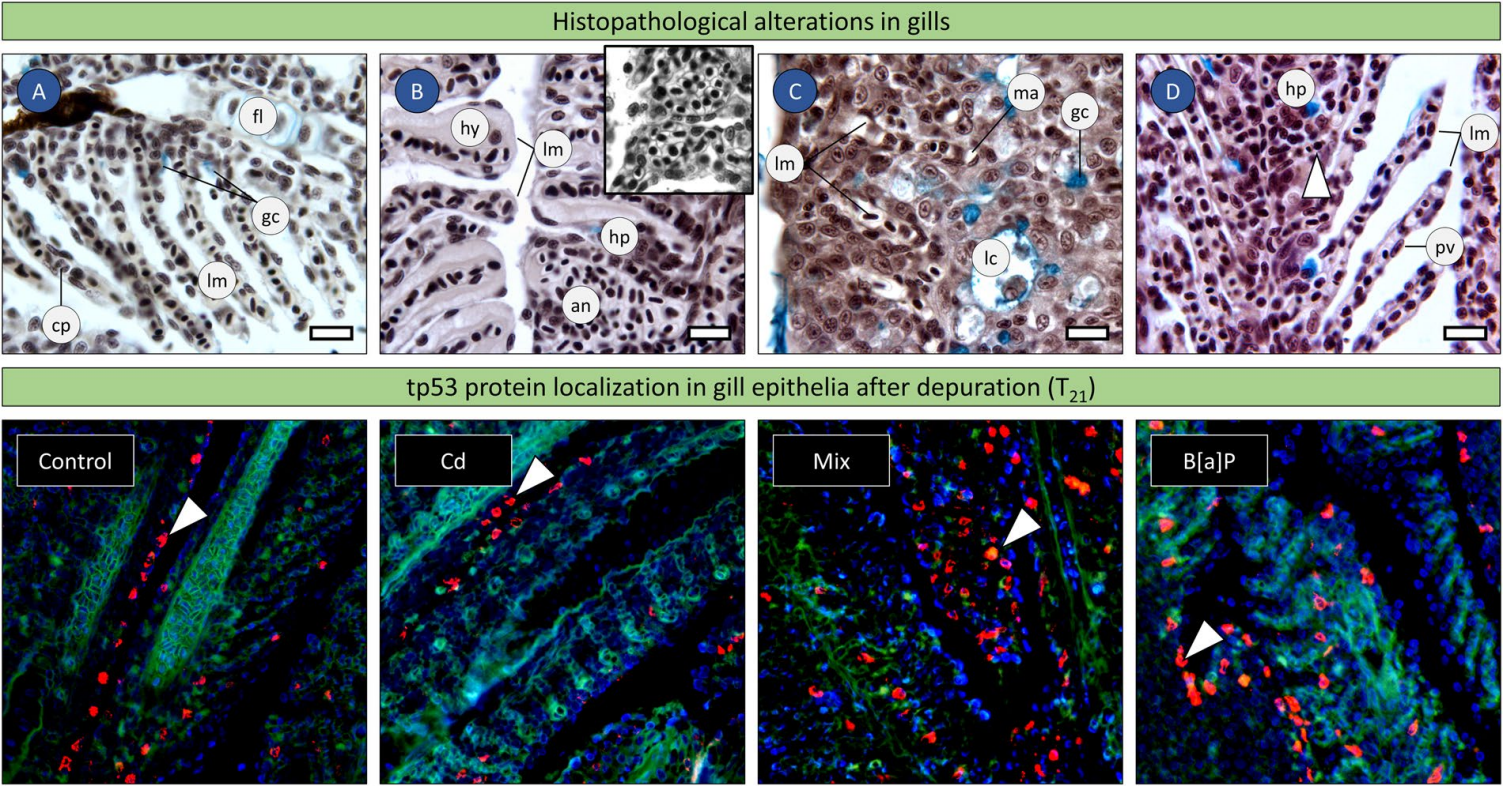
Histopathological evaluation of tested zebrafish. (**A**–**D**) Exemplificative micrographs of alterations in the gills after 14 days of exposure (AB and Van Gieson’s elastic). (**A**) Control fish. (**B**) Fish exposed to Cd, evidencing focal interlamellar hyperplasia (hp) and hypertrophy of pavement cells (hy) as main alterations, together with localised hyperaemia, likely responsible for aneurysms (an) in lamellar capillaries. Inset. Evident aneurysms in the lamellae of fish exposed to B[a]P for 14 days as well. Note vessel engorgement, which implies blood pressure, therefore excluding post-mortem blood stasis. (**C**) Fish exposed to combined Cd and B[a]P showing severe interlamellar hyperplasia. Amidst hyperplasic cells, which included goblet cells as well (stained blue), are here found macrophages (ma) and lacunae (lc) holding mucus, plasma exudate and inflammatory cells. (**D**) Gills of a fish after the one-week recovery, following exposure to Cd. Lamellae present a condition similar to that of controls, albeit some evidence of hyperplasia (hp) persisting. Apoptotic epithelial cells (arrowhead) were noted in gill epithelia at hyperplastic foci. Compare to panel A. Note normal pavement cells of lamellae. cp) blood capillary; fl) filament; gc) goblet cell (mucocyte); lm) lamellae, pv) pavement cell. Scale bars: 25 µm. Panels below: fluorescence immunolocalization of tp53 (red fluorescent probe) in gills of treated fish after the one-week recovery period, counterstained with DAPI. The signal for tp53 protein in Cd-exposed animals (arrowhead) after recovery is essentially similar to that of controls. The response was higher in animals previously exposed to B[a]P or to the mixture treatment. This was reflected in larger and denser staining spots, as well as in a broader distribution of tp53-positive cells.

All treatments yielded histopathological alterations relatively to controls during the exposure period. Upon screening whole-head sections of tested fish, gills were found to be the main affected organs, but the alterations were seemingly non-specific and in most cases related to inflammation, asserted by intrusion of macrophages and increased blood flow to gills and interlamellar epithelial cell hyperplasia. The alterations were focal, without a conspicuous trend between treatments after 7 days of exposure. However, at day 14, the exposure to the combination of both compounds caused the most severe and diffuse alterations, up to the generation of club gill filaments (meaning diffuse hyperplasia leading to complete filling of interlamellar spaces), as well as the most prominent inflammatory response. The alterations tended to be reverted to control scenario after recovery, particularly in animals exposed to Cd, who revealed more efficient recovery, albeit the persistence of foci of hyperplasia (Fig. 3A–D). Animals exposed to B[a]P and, moreover to the mixture of toxicants yielded more incomplete recovery.

Due to the differentiated response of *tp53* between exposure and recovery (recall Fig. 2) and the suggested importance of epithelial tissue in the storage of protein^11^, it was decided to localise tp53 in histological sections in order to ascertain its potential role as a response to chemical insult (e.g. as indicator of DNA damage and as an anti-proliferative agent).During exposure, no evidence for altered localisation and signal intensity could be noticed in zebrafish subjected to any treatment relatively to control. Conversely, fish recovered from B[a]P and mixture treatments (after one week of depuration, T_21_) consistently yielded more intense and diffuse immunolocalisation of tp53 protein in gill epithelia relatively to controls (Fig. 3, lower panels).

### Hierarchical clustering

**Figure 4:**
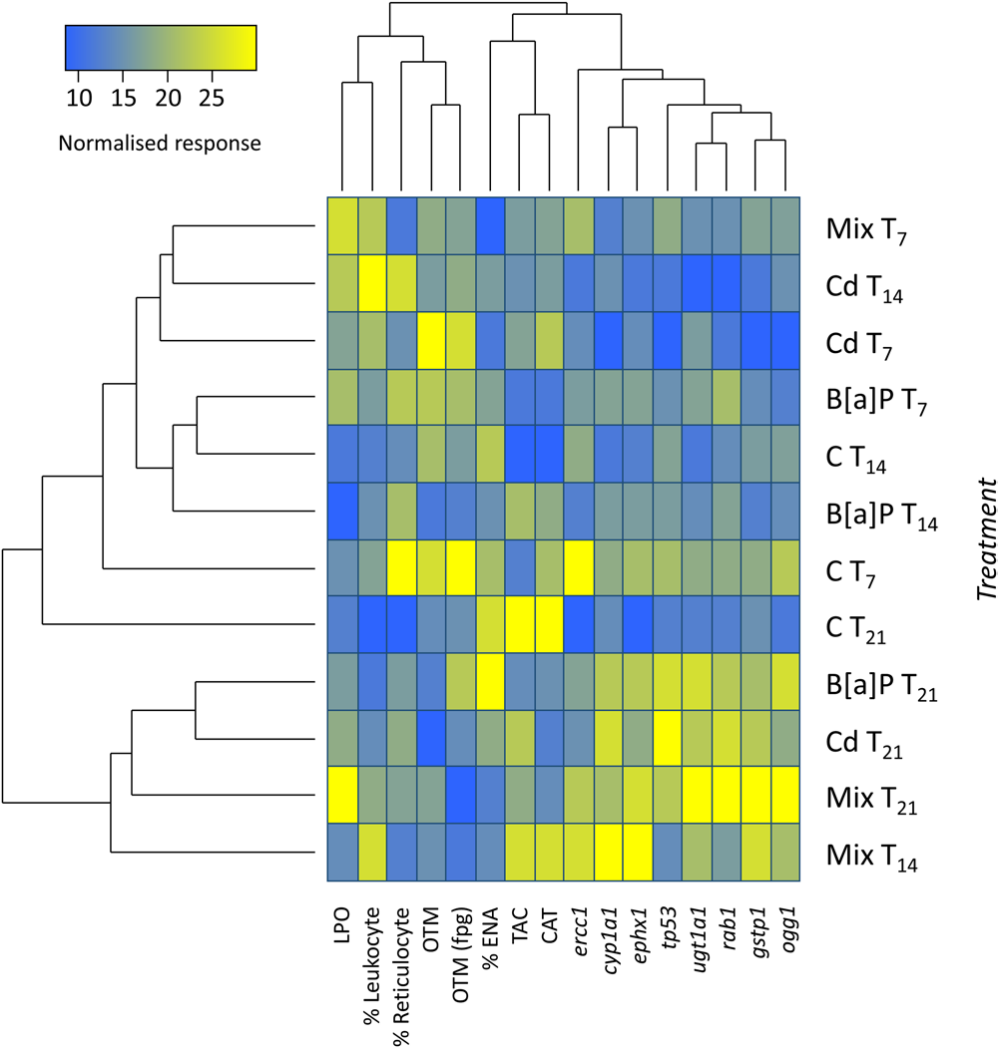
Cluster analysis integrating normalised molecular and biomarker data. Heatmap showing the relative response of all prospected biochemical and molecular endpoints (normalised data) and respective hierarchical clustering dendrograms for experimental treatments (left dendrogram) and responses (upper dendrogram). Clustering indicates similarity between Cd, Mix (Cd + B[a]P) and B[a]P treatments after 21 days (i.e. after the 7-day recovery period) plus the Mix treatment after 14 days of exposure, which appear as a separate cluster relatively to other conditions. Gene expression responses and oxidative stress parameters plus erythrocytic nuclear abnormalities (ENA) form a distinct cluster as well.

Cluster analysis integrating normalised molecular and biomarker data (Fig. 4) revealed segregation between experimental treatments (left dendrogram) and endpoints (upper dendrogram). In the first case, there is an evident separation between T_21_ animals (i.e. after depuration) exposed to isolated or mixed toxicants, to which are added fish from the combination treatment after 14 days of exposure, indicating consistency of responses between these treatments, with particular respect to elevated expression of the surveyed panel of genes (see the colour chart of the same figure). The second cluster allocates the remaining treatments and controls of all time points. Clustering between endpoints yielded two distinct clusters as well, the first comprising standard and FPG post-treatment Comet data, lipid peroxidation and blood cell sorting, which tended to be elevated in animals collected during the exposure period, albeit without a clear trend among treatments and exposure times. Interestingly, ENA is not allocated within this cluster, rather forming a sub cluster together with anti-oxidant defence parameters, namely total antioxidant capacity (TAC) and catalase activity (CAT). Within this same large cluster, several responses yielded similar patterns of expression, namely, the phase I genes *cryp1a1* and *ephx1*, the DNA repair-related genes *ercc1* and *ogg1* and *ugtp1* plus *rab1*. These latter two clusters form a distinctive cluster on their own, together with the expression of *tp53*, once again indicating consistently similar responses.

## Discussion

Most of toxicological studies focuses in single-toxicant exposure and the incidence of adverse effects are rarely considered when chemical challenge is removed. This issue is, nonetheless of particular relevance, when dealing with human carcinogens, as Cd and B[a]P, since neoplasia is essentially a chronic and systemic effect that requires time to occur and may not necessarily imply significant acute effects as a trigger. The present study showed that *ras* oncogene homologue expression and epithelial cell hyperplasia, usually linked to hallmarks of cancer like resistance to cell death and cell proliferation signalling, respectively^2^, may occur after removal of toxicological challenge in a scenario of mixed carcinogens (Cd + B[a]P), even when genotoxicity was overall low during exposure. In addition, increased *tp53* expression was detected after depuration, likely as a counter-response to cell proliferation and DNA damage. Still, the changes in the expression of genes related to DNA repair, *ercc1* and *ogg1*, were not so evident throughout the experiment, which is accordant with the reduced level of DNA strand breaks detected by the Comet assay. This suggests caution when quantifying DNA strand breakage and transcriptional profiles of DNA repair genes as early-warning signals of exposure to carcinogens.

Even though neoplasia was not detected within the time-frame of the experiment, as expected, the findings indicate persistent effects of the toxicants and their mixture after depuration. This happened even in a scenario where the relatively low concentrations of either toxicant in water, single or combined, would indicate null risk of occurring significant adverse effects. This information, together with the overall trend to elevate transcriptional responses of genes related to phase I and phase II of detoxification during the depuration period, suggests a delayed response. Still, the effects noted during latter stages (2-week) of co-exposure to mixture, indicate increased function of the Aryl hydrocarbon receptor pathway (for which B[a]P is a known agonist) comparatively to exposure to the isolated toxicants.

It is likely that inflammation during the exposure period led to reduced tp53 response observed immunochemically in gills and through RT-PCR in the liver, promoting leukocyte infiltration, together with hyperplasia, which attained highest severity in animals exposed to the mixture. In accordance, the NF-kB pathway, which is pivotal in the onset of chronic inflammation and promotion of cell division, is known to be antagonist with that of tp53 (yet another transcription factor, whose expression is ubiquitous in early life stage zebrafish, when cell proliferation is high^12^). This may partially explain why, once insult was retracted, i.e., during depuration, tp53 protein levels were immunohistochemically found to be increased in gill epithelia of animals subjected to all toxicants, albeit especially in animals previously exposed to B[a]P and, moreover, to the combination of B[a]P with Cd. The transcription of *tp53* in the liver yielded similar results but exposure to Cd caused the highest increase. In part, these differences might be due to the fact that the activity of tp53 is triggered by many stress factors (including DNA damage and oncogene activation) and plays anti-inflammatory and anti-cell proliferation roles^13,14^, being an antagonist of *ras*^15^. Also, differences between hepatic and gill responses should be safeguarded, as there may exist organ-specific pathways.

Genotoxic events were low in the present circumstances of assessment, i.e., involving ecologically-relevant concentrations of Cd, B[a]P or their combination, and time-responsiveness was elusive. On aggregate, the present findings indicate that, PAH metabolism and downstream effects like hyperplasia, inflammation and, at the molecular level, *rab1* and *tp53* over-expression does not necessarily mean preceding acute DNA damage. Additionally, reduced oxidative stress, indicated by unaltered lipid peroxidation and catalase activity, can relate to a low incidence of DNA damage via oxidative radicals. It must be noted, though, that the relation between the amount of DNA damage and oncogene expression is not entirely understood. In fact, Shive *et al*.^16^ linked the expression of a human *ras*-family oncogene in the gills of transgenic zebrafish with genotoxicity-independent inflammation and gill epithelial cell hyperplasia, similarly to the present work, hypothesizing that mutagenesis is the key element needed to trigger neoplasia. Also, Wei *et al*.^17^ disclosed that the amount of *ras* mutations caused by B[a]P is not dose-dependent in mouse skin tumours. Also, overexpression of *Ras*-family oncogenes in human epithelial cells (skin, in the case) has been linked with their clonogenic potential^18^, which forms a link to hyperplasia, as oncogenes encode essentially mitogenic and pro-survival factors. Accordingly, oncogene expression may be especially promoted in fish gill epithelial cells which are known to have stem cell-like behaviour, which enables, inclusively, immortalization *in vitro*^19^. Altogether, genotoxicity increases the probability of occurring mutations but oncogene activation may not require extensive DNA damage, in large part due to the affinity of PAH metabolites toward promoter regions of *Ras* oncogenes^20^, which is mostly co-adjuvated by inflammation-promoted cell proliferation, regardless if inflammatory events occur as direct response to chemical insult or to the injury that follows. Consequently, it may be inferred that mutation burden and transcriptional profiling should be interpreted independently in cancer research at least until a clear link between the two is not determined, which may vary with carcinogen as well as with type of cancer and its development stage.

There have been other works that showed, in several animal models, that the *tp53* response is intricate and not linearly related to toxicologically challenge, as in the present study. Luzio *et al*.^21^, for instance, found *tp53* gene expression in zebrafish gills to be elevated following exposure to Cu, albeit not in a dose- or time-dependent manner, as animals subjected to the highest concentration 100 µg L^−1^ yielded a more reduced response and decreasing from just a few hours of exposure onwards. Altogether, the same authors, who focused on the induction of apoptosis, proposed a model for the crosstalk between caspase-dependent and AIF-dependent (apoptosis inducing factor) programmed cell death in gills. This highlights that the tp53 response, which is well conserved among vertebrates, counterbalances mitogens like oncogenes and promotes apoptosis when subcellular damage rises above a critical threshold (see den Hertog^22^, for a recent dedicated review on zebrafish tp53). However, the role of tp53 protein in neoplasic disease is far more complex than is activation as an anti-proliferative agent, as mutations in the gene encoding this tumour suppressor are linked to a large percentage of malignancies. In fact, it has been highlighted that, for instance, in smoking-induced lung cancer, the number of *tp53* mutations is increased^23^, leading to its inactivation. Interestingly, tobacco smoke allocates Ahr agonists, like B[a]P, and heavy elements such as Cd. Altogether, *tp53* expression may result from a balance between mutation, the complex mechanisms that mediate inflammation and the expression of pro-mitogens, such as *rab1*.

It is acknowledged that DNA damage can be linked to neoplasic risk, precisely due to mutations in key regions of oncogenes and tumour suppressors, activating and inactivating them, respectively^24^. Besides the affinity of B[a]P diol epoxides toward specific sites, hindered DNA repair (to remove toxicant-DNA adducts), also facilitates mutation in these genes^20,25^. Cadmium, in its turn, penetrates nuclei and intercalates with DNA only at extremely high concentrations. Its *modus operandi* involves interference with enzymes, with emphasis on those containing divalent metals, with which it may compete for the active centre, such as the zinc-finger proteins that take part in DNA damage detection^26,27^, thus hindering DNA repair when co-exposure to genotoxicants like B[a]P is involved^28^. The combined action of the two xenobiotics may thus lead to hampered or delayed DNA repair, potentially favouring mutagenesis even after removal of toxicological challenge, which may contribute to explain increased *rab1* and *tp53* expression at the depuration period (T_21_). Still, even though the tp53 protein has been suggested as a biomarker of exposure to environmental genotoxicants in fish^29^, there has been little effort to enforce its application in toxicity testing and biomonitoring. Interestingly, the expression of *tp53* is not linked to clastogenic and aneuplogenic events^30^, i.e. those leading to erythrocytic nuclear abnormalities, which not only contributes to explain the slight link between genotoxicity and *tp53/rab1*, at least at whole chromosome-level DNA lesions. In fact, in the present work, it was the integration of molecular and biochemical responses that yielded a more informative pattern of consequences, allowing the distinction between exposure and depuration periods and, moreover, the effects of co-exposure comparatively to the treatments with isolated toxicants. This has evident implications for the traditional biomarker-based approach for risk assessment strategies for pollutants. This issue, widely acknowledged nowadays, is leading towards integrative strategies, omics included, for the discovery of novel biomarkers or biomarker signature^6^. It must be noted, however, that the current perspectives on signature discovery, whether focusing on mutational burden of gene expression profiling, although promising, have been chiefly applied onto human cancer to identify type, stage and even risk of relapse following treatment^7,8^. Their value as early-warning signals of exposure to carcinogens and to determine risk before neoplasms are formed has still to be fully determined.

It was hereby shown that, 14 days of exposure to ecologically-relevant concentrations of the toxicants, isolated or combined, are sufficient to cause inflammatory response and gill epithelia hyperplasia. Co-exposure enhanced effects during exposure and further delayed recovery to which is added increased expression of a *ras*-family oncogene. As combined effect, cell proliferation and resistance to cell death and anti-proliferative agents (like tp53) contributed to elevate neoplasic risk even after depuration. This delay has already been pointed out in previous works dealing with acute exposure to Cd and B[a]P in fish suggesting, inclusively, hindered apoptosis of damaged cells and impaired liver tissue recovery^28^. Overall, the results suggest that the fish responded to chemical insult by activating phase I and II systems in the liver, as well as DNA repair enzymes, suggesting that the basal response mechanisms were not overwhelmed, leading to reduced oxidative and genotoxic events. Still, the combination between Cd and B[a]P tended to generate elevated effects comparatively to exposure to isolated toxicants, noted both molecularly and histopathologically. Altogether, it has been demonstrated that pathological traits associated to neoplasia can arise from short-term co-exposure to low doses of environmental toxicants, and persist after removal of insult, namely oncogene expression, inflammation and epithelial cell hyperplasia. The findings call for caution when taking single-substance environmental quality guidelines for genotoxicants, mutagens and carcinogens.

## Material and Methods

### Test Compound solutions

The cadmium solution was obtained by preparing a 10 mg mL^−1^ stock solution from a CdCl_2_ Tritisol standard (Merck, Darmstadt, Germany), diluted in Milli-Q grade ultrapure water (18.2 MΩ cm). The benzo[a]pyrene (>97% purity, Sigma, St Louis, MO, USA) solution was prepared from a 0.1 mg mL^−1^ stock diluted in 100% dimethyl sulfoxide (DMSO).

### Animals

Adult (3 month-old) female Tg(β-actin:HRAS-EGFP) strain zebrafish (*Danio rerio*) were obtained from *Instituto de Medicina Molecular* (IMM, Lisbon, Portugal) zebrafish facilities. This strain is genetically-modified to express membrane-targeted green fluorescent protein (GFP), originally developed for microscopy developmental studies^31^. Fish were acclimatized for 20 days, at a density of three fish per litre, with controlled temperature (27.3 ± 0.6 °C) and photoperiod (10 h light:14 h dark). Fish were fed twice a day with SERA flakes. All experiments conducted in the present research were carried out in strict accordance with the recommendations of the Portuguese and European Union legislation for animal experimentation following the approval of the National Directorate for Veterinary (DGVA).

### Zebrafish exposure

A semi-static assay was performed in 10 L glass aquaria in the same conditions of the rearing period. To ensure constancy of the water parameters (temperature = 26.3 ± 0.5 °C, pH 7.98 ± 0.14, ammonia = 0.6 ± 0.2 mg NH_4_ L^−1^), 50% of the total water volume was replaced every 48 h with freshly-prepared exposure medium.

Seventy-two fish were randomly distributed by four experimental treatments: Control (C), Cadmium (Cd), Benzo[a]pyrene (B[a]P) and Cd plus B[a]P combination (Cd + B[a]P). The nominal contaminant concentrations for each experimental test were 100 µg Cd L^−1^ and 500 ng B[a]P L^−1^. All experimental treatments, including the control, received the same proportion of the vehicles (ultrapure water and DMSO). The DMSO concentration in all test aquaria was 0.01% v/v throughout the assays.

Animals were exposed to contaminants for 14 days, followed by a depuration period of 7 days, counting for an overall bioassay duration of 21 days (exposure + depuration). Six animals (biological replicates) per test were collected at days 7 (T_7_), and14 (T_14_) of exposure to the toxicants and at day 21 (T_21_), which corresponds to the end of the depuration period (7 days). Blood was collected from the caudal artery (above the lateral line system) and processed immediately for genotoxicity assessment. Animals were afterwards euthanized by cervical sectioning, measured, and dissected for tissue collection. Whole hepatopancreas was excised and preserved in RNAlater (Qiagen) for RT-PCR analyses. Right gill arches were snap frozen and kept as −80 °C for biochemical analysis, whereas the remaining head portion of each animal was immediately processed for histology.

### DNA damage

Immediately after collection, blood aliquots were either diluted (1/100) in cold PBS (phosphate-buffered saline) for the Comet assay or smeared on glass microscopy slides (followed by air-drying) for the erythrocytic nuclear abnormalities (ENA) test.

The alkaline Comet assays was performed as described earlier^32^, adapted for fish erythrocytes^33^ and was combined with the formamidopyrimidine glycosylase (FPG) DNA repair enzyme according to the method described by^34^, with modifications. In brief: After the basic steps of sample collection, cell suspension, cell embedding in pre-coated slides and cell lysis (see Supplementary Table S1, for specifics), slides were briefly washed with Milli-Q water and placed in 40 mM HEPES buffer (enzyme reaction buffer, pH 8.0, containing also 0.1 M KCl, 0.5 mM EDTA and 0.2% m/v BSA), for 5 min, at 4 °C. Then, slides were treated with 2 × 30 μL of HEPES enzyme buffer alone, or with 0.11% m/v of FPG enzyme (Biorbyt, Cambridge, United Kingdom) in buffer, sealed with a coverslip and incubated in a humidified chamber (at ≈37 °C) for 25 min. Then slides were immersed in electrophoresis buffer (pH 13) during 40 min and electrophoresis was run for 30 min at 25 V, using a Bio-Rad Sub-Cell 96 apparatus. Afterwards, the slides were neutralized in 0.1 M Tris-HCl buffer (pH 7.5) for 15 min, at 4 °C. Slides were stained with ethidium bromide and analysed with a DMBL model microscope adapted for epifluorescence with an EL6000 light source for mercury short-arc reflector lamps and a N2.1 filter (all from Leica Microsystems). Approximately, 100 random comets were analysed per slide, using the CometScore 1.5 (TriTek, VA, USA) software. The Olive tail moment (OTM) was employed as metric for DNA (double and single) strand damage^35^. The results are expressed as mean OTM per individual.

### Erythrocytic Nuclear Abnormalities

Erythrocytic nuclear abnormalities (ENA) were assessed on blood smears fixed with methanol (10 min) and stained with acridine orange fluorochrome (Supplementary Table S1, for further details) as a measure of clastogenic/aneugenic genotoxic events^36^. At least 1000 mature and intact erythrocytes were scored per slide and cells with nuclear abnormalities were analysed^36–38^, using the aforementioned equipment for UV microscopy, equipped with an I3 filter (Leica Microsystems). The results are expressed as percentage (%) of mature red blood cells exhibiting ENA per individual. The percentages of immature erythrocytes (reticulocytes) and leukocytes were also scored.

### Biochemical biomarkers

Gill samples (6.33 ± 0.82 mg wet weight) were used for the biochemical analysis: (i) protein content^39^; (ii) lipid peroxides (LPO) assay using the thiobarbituric acid-reactive substances (TBARS), measured as malondialdehyde (MDA) content^40,41^; (iii) catalase activity (CAT) using H_2_O_2_ as substrate, measured as the amount of enzyme needed to form 1 nmol formaldehyde per minute^42^ adapted to 96-well microplate reader^43^; and (iv) total antioxidant capacity (TAC) using the trolox equivalent antioxidant capacity (TEAC) principle^44^. These methods are described in detail in the Supplementary S1 Table.

### RT-PCR analysis

Total RNA was extracted from pooled hepatopancreas samples (≈15 mg), due to the small size of the animals: the hepatopancreas was excised from six specimens of each experimental group and time point and pooled into two independent samples comprising three animals each. Extraction was done using the RNeasy and RNase-Free DNase Set kits (Qiagen), following manufacturer instructions. Total RNA was quantified using a NanoDrop 2000 (Thermo Scientific) and reverse-transcribed (2 µg per reaction) using the Transcriptor Kit (Roche Applied Science). Real-time PCR was performed on a LightCycler 480 II instrument using the LightCycler 480 SYBR Green I Master Mix (Roche Applied Science), using 96-well plates. Each reaction contained 20 µL of the mixture prepared with 10 µl of the Master Mix, 7 µl of PCR-grade water, plus 1 µl of each primer’s solution (at 10 µM) and 1 µl of template cDNA. Primers were either retrieved from available literature^45^ or designed for each target gene in order to obtain similar-size amplicons and annealing temperatures, based on *D. rerio* sequences deposited in public-access databases (Table 1). The gene set was selected according to their relevance in mechanisms of DNA repair (*ercc1* and *ogg1*), phases I (*cyp1a1* and *ephx1*) and II (*gstp1* and *ugt1a1*) of detoxification and neoplasia development (*rab1a*) and its counter-response (*tp53*). The oncogene *rab1a* was selected for being a zebrafish homologue of the Ras-family oncogenes, for which there is evidence that the metabolites deriving from B[a]P or similar carcinogenic PAHs have specific affinity^20^. Data were processed according to the ΔCt method, taking the expression of *ribosomal protein L13a* (*rpl13a*) as reference gene^45^. The Ct estimates were obtained with the LightCycler 480 II dedicated software. Primer melting curves were analysed to assure primer specificity. Analyses were performed in duplicate per independent sample.

**Table 1:** Sequence, primer and amplicon information for the gene expression sequence tags analysed by RT-PCR in the present study.

Gene ID	GenBank accession	Primer	Primer sequence (5′–3′)	Amplicon size (bp)	Tann (°C)
rpl13a	NM_212784	Forward	TCCCAGCTGCTCTCAAGATT	154	52
		Reverse	TTCTTGGAATAGCGCAGCTT		
cyp1a1	NM_131879	Forward	CCTGGGCGGTTGTCTATCTA	184	52
		Reverse	TGAGGAATGGTGAAGGGAAG		
ephx1	NM_201068	Forward	CCTCGATACCTGGTTACGGC	213	56
		Reverse	CAGGGGGAGCGAAGTTAATG		
gstp1	NM_131734	Forward	CTCCCTACACACTCACATAC	191	52
		Reverse	CTGAAACAGCACCAGGTCAC		
ugt1a1	GU299096	Forward	CTGGGCTCCATGGTGTCAC	208	55
		Reverse	GTGATCCACCATGTGTAAC		
ercc1	NM_001103138	Forward	TTGGAGGGCATCATTAAAGC	197	50
		Reverse	GCCTGATGGTCTCAGATGGT		
ogg1	NM_0011233082	Forward	ATGTCCCAGCATGCTCTAC	237	49
		Reverse	GTTTTCTGGTCCTGGATTG		
tp53	NM_001271820	Forward	ATCATCTGAGCCCAAACAGG	173	52
		Reverse	AAATGACCCCTGTGACAAGC		
rab1a	NM_001007161	Forward	CGATGAATCCCGAATATGAC	221	50
		Reverse	GATTGTGCGAAACCTCTCC		

### Histopathology

Due to the small size of animals that invalidated retrieving simultaneously samples for all endpoints, whole longitudinal sections of head, which permits full access to gill cavity, anterior gut and central nervous system, were fixed in Davidson’s solution, embedded in paraffin and screened for histopathological alterations. Sample staining was performed using a combination of Alcian Blue for mucosubstances and acidic sugars, van Gieson’s elastic stain for fibres, which includes Weigert’s Iron Haematoxylin^46^. Slides were qualitatively screened for histopathological alterations in all individuals per experimental condition^47^.

### Immunohistochemistry

As the findings from the histopathological approach indicated cell proliferation and clearance as an important benchmark (see Results), immunohistochemistry for fluorescence microscopy (IF) was used to provide phenotypical anchoring of molecular data. As such, tp53 was localized in paraffin sections using rabbit anti-zebrafish tp53 (polyclonal) antibody (AntibodiesOnline). The Alexa Fluor 594-conjugated secondary antibody (Invitrogen) was used for labelling. Nuclear counterstaining was done using DAPI. A Leica DMLB microscope equipped with an EL2000 light source for epifluorescence was used for all microscopy analyses.

### Statistical integration of data

Biomarker data were analysed through the non-parametric Kruskal-Wallis ANOVA by ranks *H* statistic for multiple comparisons, following the invalidation of at least one of the assumptions for parametric analysis, namely homogeneity of variances and normality, checked through Levene’s test and *p*-plots, respectively. For pairwise comparisons between tests and respective controls at each sampling time the non-parametric, Mann-Whitney *U* test was employed. The Wilcoxon’s Matched Pairs test was applied to compare data from the standard and FPG-modified Comet assay. Statistical analyses were performed with Statistica (Statsoft Inc., Tulsa, OK, USA)^48,49^.

Gene expression data were analysed through analysis of variance, namely *F* tests and Tukey’s Honest Significance difference (HSD) test for effect testing and multiple comparisons, respectively. Data were hitherto log-transformed to meet assumptions for parametric ANOVA. Biomarker and gene expression data were integrated using hierarchical clustering on a quantile-normalised endpoint *versus* experimental treatment data matrix^50^. Complete linkage and Euclidean distances were employed as amalgamation rule and metrics, respectively, to draw association dendrograms. Statistics were performed using R^51^. The significance level was set at α = 0.05 for all statistical procedures.

## Electronic supplementary material

Supplementary information
