# Supplementary material for: Co-exposure to environmental carcinogens in vivo induces neoplasia-related hallmarks in low-genotoxicity events, even after removal of insult
Source: Sci Rep. 2018 Feb 26;8:3649. doi: 10.1038/s41598-018-21975-w (PMC5827018; doi:10.1038/s41598-018-21975-w)
Supplement: Supplementary file 1 — Supplementary information [file 41598_2018_21975_MOESM1_ESM.pdf]

SUPLEMMENTARY INFORMATION

Co-exposure to environmental carcinogens *in vivo* induces neoplasia-related hallmarks in low-genotoxicity events, even after removal of insult

(Total 10 pages, with one table and 4 figures)

Marta Martins[1,21], Ana Silva[1], Maria H. Costa[1], Célia Miguel[3,4,5], Pedro M. Costa[1,6]

[1]MARE - Marine and Environmental Sciences Centre, Departamento de Ciências e Engenharia do Ambiente, Faculdade de Ciências e Tecnologia da Universidade Nova de Lisboa, 2829-516 Caparica, Portugal.

[2]UCIBIO, REQUIMTE, Departamento de Química, Faculdade de Ciências e Tecnologia, Universidade Nova de Lisboa, 2829-516 Caparica, Portugal.

[3]Instituto de Biologia Experimental e Tecnológica/Instituto de Tecnologia Química e Biológica, Universidade Nova de Lisboa (IBET/ITQB-UNL), Apartado 12, 2781-901 Oeiras, Portugal

[4]Instituto de Tecnologia Química e Biológica António Xavier, Universidade Nova de Lisboa (ITQB NOVA), Av. República, 2780-157, Oeiras, Portugal

[5]Biosystems & Integrative Sciences Institute, Faculdade de Ciências, Universidade de Lisboa (FCUL), Campo Grande, 1749-016 Lisbon, Portugal

[6]UCIBIO-REQUIMTE – Research Unit on Applied Molecular Biosciences, Departamento de Ciências da Vida, Faculdade de Ciências e Tecnologia da Universidade Nova de Lisboa, 2829-516 Caparica, Portugal.

[1] Corresponding author.

Phone. +351 212 948 300 ext.10103

Fax. +351 212 948 554

e-mail: marta.martins@fct.unl.pt (M. Martins)

CONTENTS

S1 Table.

Methodology details .... p. S3

S1 Figure.

Common type of cells and erythrocytic nuclear abnormalities (ENA) observed in exposed *Danio rerio* .... p. S6

S2 Figure.

Average percentage of mature erythrocytes exhibiting ENA per treatment and sampling time .... p. S7

S3 Figure.

Mean percentage of reticulocytes and leukocytes in tested fish per treatment and sampling time .... p. S8

S4 Figure.

Average biochemical biomarker (LPO, CAT, TAC) results in the gills of *Danio rerio* per treatment and sampling time .... p. S9

References p.S10

Table S1. Methodology details

**Biomarker**	**Detailed description**
**Alkaline Comet assay**<br>(Singh et al., 1988, Costa et al., 2008; Martins & Costa, 2015)	Cell suspensions (20 uL) was diluted in 180 uL of melted (35-40 ºC) 1% (w/v) low-melting point agarose (LMPA, Sigma) prepared with PBS. Aliquots of the cell suspension (2×75 uL) were placed in glass microscopy slides previously coated with 1% (w/v) normal melting-point agarose (NMPA, Sigma) prepared in TAE buffer. After agarose solidification (15 min, 4 ºC, in dark), slides were dipped for 1 h in cold (4 ºC, in dark) lysis solution (2.64% NaCl w/v, 3.72% EDTA w/v and 5 mM TRIS) to which 10% (v/v) DMSO and 1% (v/v) Triton-X 100 were added just before use.<br>Afterwards, the slides were briefly washed with Milli-Q water and placed in 40 mM HEPES buffer (enzyme reaction buffer, pH 8.0, containing also 0.1 M KCl, 0.5 mM EDTA and 0.2% m/v BSA), for 5 min, in cold. Then, slides were treated with 2×30 µL of HEPES enzyme buffer alone, or with 0.11% m/v of FPG enzyme (Biorbyt, Cambridge, United Kingdom) in buffer, sealed with a coverslip and incubated in a humidified chamber (at ≈37ºC) for 25 min. DNA unwinding and enhanced expression of alkalilabile sites were promoted by immersing slides for 40 min (4º C, in dark) in electrophoresis buffer (pH 13). Electrophoresis was run during 30 min, at 25 V. Afterwards, the slides were neutralized in 0.1 N Tris-HCl buffer (pH 7.5) for 15 min. Slides were stained with ethidium bromide for 5 min and analysed with a DMBL model microscope adapted for epifluorescence with an EL6000 light source for mercury short-arc reflector lamps and a N2.1 filter (all from Leica Microsystems). Approximately, 100 random comets were analysed per slide, using the CometScore 1.5 (TriTek, VA, USA) software. Olive tail moment (OTM) was employed as metric for DNA (double and single) strand damage (Olive, 1990).
**Erythrocytic nuclear abnormalities (ENA)**<br>(Costa & Costa, 2007)	After fixation in methanol for 15 min, the blood smears were stained with acridine orange solution 0.01% m/v AO base (Sigma, St Louis, MO, USA; maximum absorbance 488 nm) diluted in Milli-Q water, to which was added 1% v/v glacial acetic acid 0.1 g L-1 acridine orange (Sigma).After 45-60 min in the dark, slides were briefly rinsed in acid alcohol (0.5% v/v glacial acetic acid in absolute ethanol) and then dehydrated in absolute ethanol for 30 s - 1 min. Afterwards, slides were allowed to dry (in the dark) and mounted with DPX resinous medium (BDH, Poole, England). At least 1000 mature and intact erythrocytes per slide were scored to

	determine the percentage of cells with nuclear abnormalities using a UV microscopy, equipped with an I3 filter (also from Leica Microsystems).
**Lipid peroxides (LPO)**<br>(Uchiyama and Mihara, 1978, adapted to microplate reader by Costa et al. 2011)	Cold trichloroacetic acid (10% m/v) was added to homogenates (1:1), which were then incubated for 15 min at 4 ºC to allow deproteination. The samples were then centrifuged for 10 min at 9000 × *g*. Following this step, the supernatant was mixed with an equal volume of cold thiobarbituric acid (1% m/v) followed by incubation for 10 min in boiling water. Samples were afterwards placed on ice to stop the reaction. The red pigment was extracted with 250 µL pyridine/butanol mixture (1/15, v/v) per 240 µL of the reaction, following mixing and centrifugation for 2 min at 16000 × *g* to separate the aqueous and organic phases. The supernatants were loaded (2 × 100 µL) into 96-well microplates and the absorbance measured at 532 nm. Malondialdehyde bis(dimethylacetal) (MDA), from Merck (Darmstadt, Germany) was used as standard in order to obtain an eight-point calibration curve.
**Catalase activity (CAT)**<br>(Johansson & Borg, 1988, adapted to 96-well microplate reader by Madeira et al., 2015)	Homogenates (20 µL) were transferred to the microplates' wells followed by adding 100 µL of assay buffer (containing potassium phosphate, 100 mM, pH 7.0), 30 µL of absolute methanol and 20 µL of hydrogen peroxide (0.035 M), to initiate the reaction. The mixture was allowed to incubate for 20 min in the dark, at room temperature, with gentle mixing. After this step, the reaction was stopped by adding 30 µL of KOH (10 M) and 30 µL of Purpald (34.2 mM), followed by a 10-min incubation period in the shaker. Finally, 10 µL of potassium periodate (65.2 mM) was added and the absorbance was read at 540 nm. Bovine liver catalase solution (from Sigma) was employed as positive control. Formaldehyde (4.25 M) was used to obtain an eight-point standard calibration curve for quantification.
**Total antioxidant capacity (TAC**)<br>(Miller et al., 1993)	In each well microplate, 10 µL of each sample was mixed with an equal volume of 90 µM myoglobin. Afterwards, 150 µL of 600 µM 2,2'-azino-bis(3-ethylbenzthiazoline-6-sulfonic acid) (ABTS) were added to the mixture, followed by 40 µL of 500 µM hydrogen peroxide to initiate the reaction. The absorbance was measured at 410 nm. A calibration curve was obtained from seven Trolox (Sigma) standards.
**Total Protein**<br>(Bradford, 1976)	180 µL of Bradford reagent (Comassie Blue G250, methanol, phosphoric acid, distilled water) were added to 10 µL of each sample. The assays were performed in triplicate. The absorbance was read at 595 nm. BSA standards were used for a calibration curve. Total protein measurements were used to normalize all biomarker levels.

**Histopathology**<br>(Costa & Costa, 2012; Martins et al., 2016)	Whole longitudinal sections of head, which permits full access to gill cavity, anterior gut and central nervous system, were immediately immersed in Davidson's fixative solution (10% v/v formaldehyde, 30% v/v ethanol and 10% v/v glacial acetic acid) for c.a. 24 h at room temperature. Samples were afterwards dehydrated in a progressive series of ethanol (70, 96 and 100% v/v), intermediately infiltrated in xylene and embedded in molten paraffin. Samples were then sectioned at 5 μm thickness using a Jung RM 2035 model microtome (Leica Microsystems) and collected in order to obtain at least eight serial sections per slide. Two slides of each animal were deparaffinated with xylene, rehydrated and stained combining Alcian Blue (40 min) for mucosubstances and acidic sugars, van Gieson's elastic stain (Acid Fuchsin and Picric Acid, for 6 min) for fibres and Weigert's Iron Haematoxylin (10 min) as counterstain. All intermediate washing steps were done with Milli-Q grade ultrapure water (18.2 MΩcm) to remove excessive dyes. Slides were afterwards dehydrated, cleared with xylene and mounted with DPX resin. Slides were qualitatively screened for histopathological alterations in all individuals using a DMLB model microscope (Leica Microsystems).
**Fluorochrome immunohistochemistry**	Three slides of each treatment were deparaffinised with xylene, rehydrated and immersed in Dulbecco's PBS for 6 min. Afterwards, slides were treated with 200 μL of 0.1% Trypsin prepared in PBS to promote antigen retrieval, sealed with a coverslip. After 15 min, the slides were washed twice with PBS. Afterwards, four drops of Tissue enhancer (Thermo) were placed in each slide and after 30 min of incubation, the coverslip was removed and the slides were washed twice with PBS. Two slides of each experimental treatment were incubated overnight with 200 μL of rabbit anti-zebra primary antibody (AntibodiesOnline) diluted to 1:50 with blocking solution (2% BSA in PBS with 0.1% TritonX-100). A negative control was also performed adding 200 μL of PBS to one slide of each treatment. After the removal of coverslip, the slides were washed with PBS and incubated with 200 μL of the fluorochrome-labelled Alexa Fluor 594-conjugated secondary antibody (Invitrogen) previously diluted to 1:100 with blocking solution, for 2h, in dark. Afterwards, four drops of aqueous mounting agent DAPI were added to each slide, in dark and immediately analysed using a Leica DMLB microscope equipped with an EL2000 light source for epifluorescence. All incubation steps were performed in a covered humidified chamber.

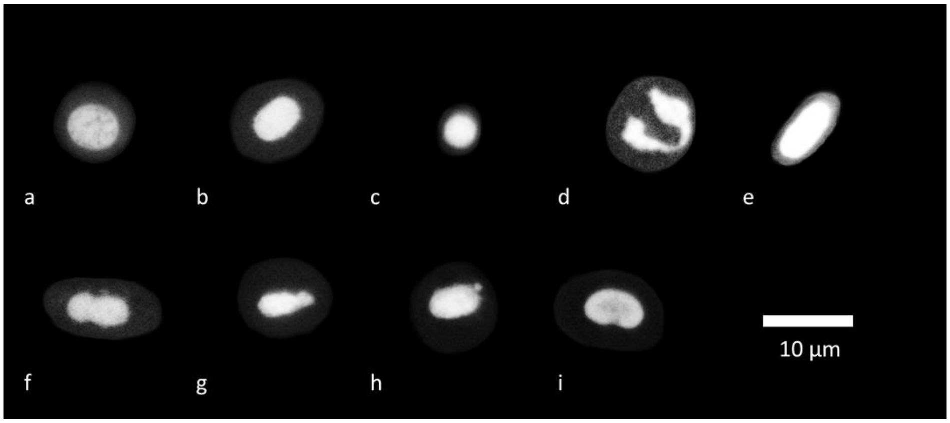

Figure S1. Common type of cells (a-e) and erythrocytic nuclear abnormalities (ENA) (f-i) observed in exposed *Danio rerio*: (a) reticulocyte , (b) mature erythrocyte, (c) lymphocyte, (d) neutrophil, (e) thrombocyte, (f) bilobed nucleus, (g) budding nucleus, (h) micronucleus (arrow), (i) kidney-shaped nucleus.

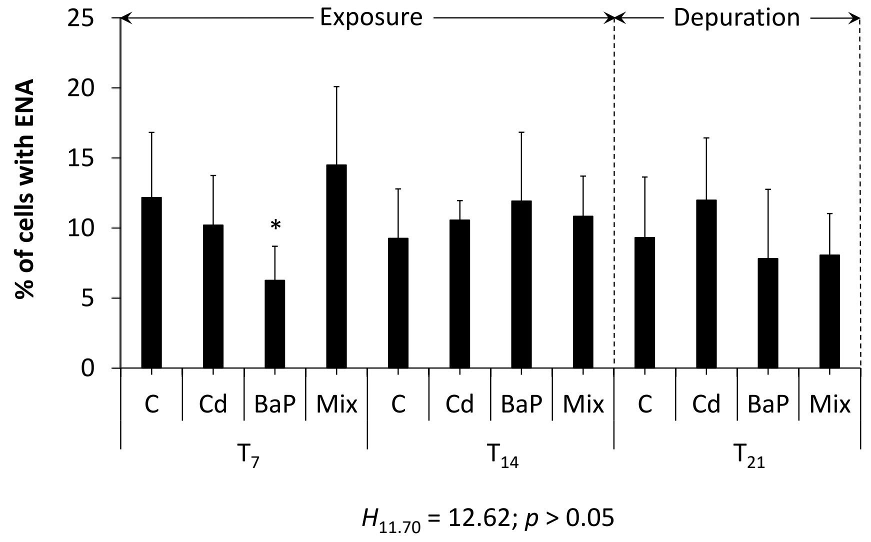

Figure S2. Average percentages of mature red blood cells exhibiting erythrocytic nuclear abnormalities (% of cell with ENA) in female zebrafish (*Danio rerio*) subjected to control treatment (C); 100 μg L^{-1} cadmium (Cd); 500 ng L^{-1} benzo[*a*]pyrene (B[*a*]P) or combined toxicants (Mix), comprising 100 μg L^{-1} Cd plus 500 ng L^{-1} B[*a*]P. Sampling times were scheduled for days 7 (T_7), 14 (T_{14}) and 21 (T_{21}). Error bars represent 95% confidence intervals. * indicates significant differences to respective sampling time control (Mann-Whitney *U* test, $p < 0.05$).

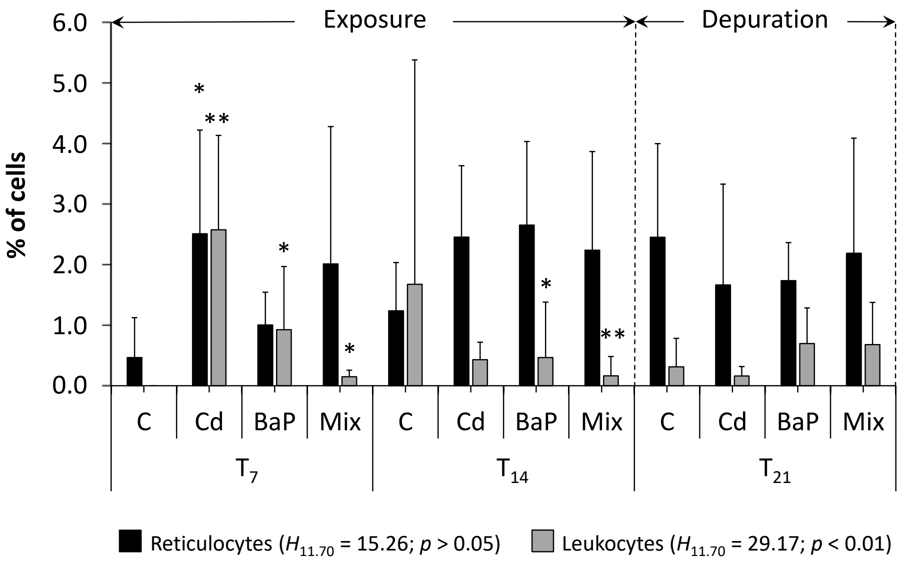

Figure S3. Mean percentage of reticulocytes and leukocytes in tested fish. (C – control; Cd – cadmium treatment (100 µg L^{-1}); B[*a*]P – benzo[*a*]pyrene treatment (500 ng L^{-1}); Mix – combined Cd and B[*a*]P (100 µg L^{-1} plus 500 ng L^{-1}, respectively). Sampling was scheduled for days 7 (T7), 14 (T14) and 21 (T21). Error bars reveals 95% confidence intervals. * and ** indicate significant differences to control, $p < 0.05$ and $p < 0.01$, respectively (Mann-Whitney *U* test).

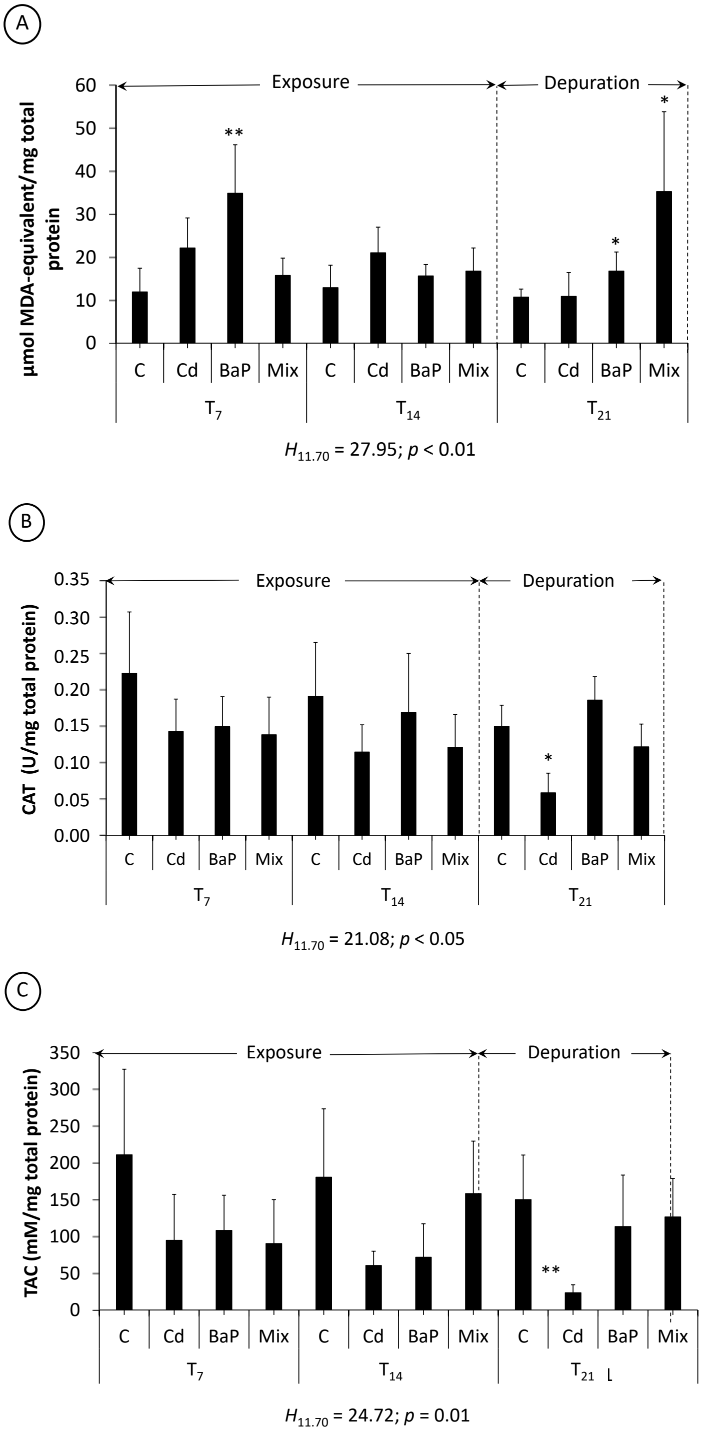

Figure S4. Average biochemical biomarker results in the gills of female zebrafish (*Danio rerio*) subjected to control treatment (C); 100 µg L^{-1} cadmium (Cd); 500 ng L^{-1} benzo[*a*]pyrene (B[*a*]P) or combined toxicants (Mix), comprising 100 µg L^{-1} Cd plus 500 ng L^{-1} B[*a*]P. Sampling times were scheduled for days 7 (T_7), 14 (T_{14}) and 21(T_{21}). A) Lipid peroxidation, measured through the TBARS protocol (MDA-equivalent per total protein); B) catalase activity (CAT); C) total antioxidant capacity (TAC). Error bars indicate 95% confidence intervals. * and ** indicate significant differences to respective control, $p < 0.05$ and $p < 0.01$, respectively (Mann-Whitney *U* test).

REFERENCES

Bradford, M. A rapid and sensitive method for the quantification of microgram quantities of protein utilizing the principle of protein-dye binding. *Anal. Biochem*. **1976**, 72, 248-254.

Costa P. M.; Costa M. H. Genotoxicity assessment in fish peripheral blood: a method for a more efficient analysis of micronuclei. *J. Fish Biol.* **2007**, 71A, 148-151.

Costa, P. M.; Lobo, J.; Caeiro, S.; Martins, M.; Ferreira, A. M.; Caetano, M.; Vale, C.; DelValls, T. À.; Costa, M. H. Genotoxic damage in *Solea senegalensis* exposed to sediments from the Sado Estuary (Portugal): effects of metallic and organic contaminants. *Mutat. Res.* **2008**, 654, 29–37.

Costa, P. M.; Neuparth, T.; Caeiro, S.; Lobo ,J.; Martins, M.; Ferreira, A. M.; Caetano, M.; Vale, C.; DelValls, T. Á.; Costa, M. H. Assessment of the genotoxic potential of contaminated estuarine sediments in fish peripheral blood: laboratory versus *in situ* studies. *Environ. Res.* **2011**, 111, 25-36.

Costa, P. M., Costa, M. H. Development and application of a novel histological multichrome technique on whole–body clam histopathology. *J. Invertebr. Pathol.* **2012**, 110, 411–414.

Johansson, L. H.; Borg, L. A. H. A spectrophotometric method for determination of catalase activity in small tissue samples. *Anal. Biochem.***1988**, 174, 331–336.

Madeira, C.; Madeira, D.; Vinagre, C.; Diniz, M. Octocorals in a changing environment: Seasonal response of stress biomarkers in natural populations of *Veretillum cynomorium*. *J. Sea. Res*. **2015**, *103*, 120-128.

Martins, M.; Costa. P. M. The Comet assay in Ecological Risk Assessment of marine pollutants: applications, assets and handicaps of surveying genotoxicity in non-model organism – Review. *Mutagenesis*. **2015**, *30* (1), 89-106.

Martins, M.; Santos, J. M.; Costa, M. H.; Costa, P. M. Applying quantitative and semi-quantitative histopathology to address the interaction between sediment-bound polycyclic aromatic hydrocarbons in fish gills. *Ecotox. Environ. Safe*, **2016**, 131, 164-71.

Miller, N. J., Rice-Evans, C., Davies, M. J. A new method for measuring antioxidant activity. *Biochem. Soc. T.* **1993**, 21, 95S.

Olive, P. L., Banáth, J. P., Durand, R. E. Heterogeneity in radiation-induced DNA damage and repair in tumor and normal cells measured using the “comet” assay. *Radiat. Res*. **1990**, 122, 86-94.

Singh, N. P., McCoy, M. T., Tice, R. R., Schneider, E. L. A simple technique for quantitation of low levels of DNA damage in individual cells. *Exp. Cell Res.* **1988**, 175, 184-191.

Uchiyama M, Mihara M. Determination of malonaldehyde precursor in tissues by thiobarbituric acid test. *Anal. Biochem*. **1978**, 86, 271-278.
